# Under Surveyed and Under Pressure: Additional Biodiversity Uncovered in the Freshwater Mussels (Unionida: Velesunioninae) of North‐Western Australia

**DOI:** 10.1002/ece3.73746

**Published:** 2026-06-01

**Authors:** Angus D' Arcy Lawrie, Jake Ryan Daviot, Adam Harman, Christopher Hofmeester, Joel Huey

**Affiliations:** ^1^ Trace and Environmental DNA (TrEnD) Lab, School of Molecular and Life Sciences Curtin University Bentley Western Australia Australia; ^2^ Centre for Sustainable Aquatic Ecosystems, Harry Butler Institute Murdoch University Murdoch Western Australia Australia; ^3^ Lateral Environmental West Perth Western Australia Australia; ^4^ Biologic Environmental Osborne Park Western Australia Australia

**Keywords:** freshwater mussels, Hyriidae, *Microdontia*, rivers, species delimitation, taxonomy *Alathyria*, *Westralunio*

## Abstract

Accurate species delimitation underpins freshwater biodiversity assessment and conservation, yet taxonomic uncertainty remains a major impediment for many invertebrate groups. Velesunioninae (Unionida; Hyriidae) constitutes the most diverse group of Australian freshwater mussels but is poorly documented in the Kimberley and Pilbara regions of north‐western Australia, where only three species have been reported across an area spanning ~930,000 km^2^. These distributions have historically been inferred from shell morphology, despite evidence that shell characters can overlap among species and that genetic data have identified species complexes elsewhere in Australia. Here we integrate genetic and morphological data to test for the presence of undescribed species within Velesunioninae collected from five drainages across north‐western Australia. Phylogenetic analyses of multi‐locus molecular data (*COI*, *16S*, *28S* and *18S*) identified five discrete lineages, including one described species ( *Lortiella froggatti* ), one previously recognised undescribed lineage (*Velesunio* ‘sp. Lineage A’) and three previously unknown lineages. Except for *L. froggatti* which displayed a distinct shell morphology, morphological characters were unreliable in distinguishing *Velesunio* lineages. *Velesunio* ‘sp. Lineage A’ and *L. froggatti* were identified across large geographic distances (1320 km and 550 km, respectively), while the three novel *Velesunio* lineages showed more restricted distributions. Overall, our results suggest that the apparently widespread morpho‐species previously reported from north‐western Australia may instead comprise complexes of both broadly distributed and geographically restricted undescribed taxa. Further targeted genetic surveys will be necessary to clarify species distributions and support conservation management of freshwater mussels in a region increasingly affected by water extraction and mineral development.

## Graphical abstract

Here, we tested for cryptic diversity within freshwater mussels in north‐western Australia and identified multiple undescribed lineages. Distributional patterns were complex with some taxa displaying evidence of potentially recent long‐distance dispersal whereas others were more restricted in their distributions. Overall, these findings suggest that the current reported distributions of freshwater mussels in north‐western Australia are likely misleading.

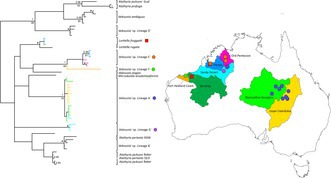

## 1 Introduction

Effective ecosystem management is contingent on the ability to classify organisms (McNeely 2002) yet only a fraction of global biodiversity is believed to be accounted for (Mora et al. 2011). This is especially the case in hyper‐diverse invertebrate groups that have received a comparatively lower proportion of research effort than vertebrates (Troudet et al. 2017). Freshwater aquatic invertebrates are at disproportionate risk of extirpation given inland freshwater environments are some of the most threatened ecosystems globally (Dudgeon et al. 2006; Reid et al. 2019). The lack of basic taxonomic information is a significant impediment to our understanding of the conservation status of many invertebrates, despite some groups displaying concerning levels of decline and, in some cases, extinctions (Strayer 2006).

Freshwater mussels (Unionida) are important ecosystem engineers of freshwater environments (Vaughn 2018). They improve water quality by filtering out algae, bacteria and suspended solids, contribute to the recycling and storage of nutrients and provide crucial habitat for co‐inhabiting aquatic species (Daviot et al. 2025; Vaughn 2018). However, freshwater mussels are particularly susceptible to environmental disturbances due to biological traits including delayed sexual maturity, sedentary adults and environmentally sensitive juvenile/larval stages (Benson et al. 2021; Böhm et al. 2021). These characteristics, combined with global declines in freshwater ecosystems, have made mussels one of the most threatened animal groups (Lopes‐Lima et al. 2018). A higher proportion of described mussel species are listed on the IUCN Red List than mammals, birds or reptiles, and current estimates suggest that 44% of all described species are of conservation concern (Lopes‐Lima et al. 2018).

Although relatively well taxonomically characterised in the northern hemisphere, comparably less is known about the freshwater mussels from the southern hemisphere (Graf and Cummings 2021). The majority of Australian Unionida belong to the Velesunioninae (Hyriidae) which currently contains the five genera: *Velesunio*, *Alathyria*, *Westralunio*, *Microdontia* and *Lortiella* (Klunzinger et al. 2023). A total of 13 described species occur in Australia with *Velesunio sentaniensis* (Haas, 1924), *Microdontia anodontaeformis* (Tapparone Canefri, 1883), *Westralunio flyensis* (Tapparone Canefri, 1883) and *Westralunio albertisi* Clench, 1957 reported from Papua New Guinea (Graf and Cummings 2021). Historically, differences in external morphological characters including beak and shell sculpture have been used to distinguish genera and species (Walker et al. 2014). However, these characters have been observed to vary within and between different species depending on local environmental factors, complicating the identification of some species from morphology alone (Baker et al. 2003, 2004; Sheldon 2017). Further, genetic data have suggested that *Velesunio* and *Alathyria* are paraphyletic and require taxonomic revision (Baker et al. 2004).

The diversity and distribution of Velesunioninae in the Kimberley and Pilbara regions (hereafter north‐western Australia) have been particularly poorly characterised, with currently only three Velesunioninae species reported from this ~930,000 km^2^ area: *Velesunio wilsonii* (Lea, 1859), *Velesunio angasi* (Sowerby, 1867) and *Lortiella froggatti* (Iredale 1934) (Kirkendale et al. 2022; Klunzinger et al. 2013; Ponder and Bayer 2004). Available distributional data suggest that *V. wilsonii* , *V. angasi* and *L. froggatti* have broad distributions, occurring across multiple drainage basins within north‐western Australia (Ponder et al. 2024). Such broad, multi‐drainage distributions are unusual when compared with several other freshwater taxa in the region, including fishes (Pepper and Keogh 2014) and caridean shrimps (Cook et al. 2011), where discrete drainage basins or river catchments are frequently associated with genetically isolated populations or distinct species (Huey et al. 2014).

Molecular data have been central to identifying these speciation patterns in other freshwater groups and have repeatedly revealed undescribed biodiversity within morphologically conserved, geographically widespread species (Pepper and Keogh 2014). Whether this pattern applies to Velesunioninae in north‐western Australia remains unclear because, with the exception of *L. froggatti* (Graf et al. 2015), there are no publicly available genetic data for specimens collected from this region. Elsewhere in Australia, molecular studies have demonstrated that identifying species via only external shell morphology can underestimate diversity within the Velesunioninae, with genetically distinct lineages exhibiting subtle (Klunzinger et al. 2021, 2022) or inconclusive (Baker et al. 2003, 2004) morphological differences. This includes the morphotype currently recognised as *V. wilsonii* that is apparently common in north‐western Australia but has been shown to comprise at least three distinct genetic lineages in eastern Australia (Baker et al. 2003). Therefore, it is unclear whether current biodiversity estimates accurately reflect the true diversity of Velesunioninae in north‐western Australia. This is concerning given the significant mineral resource developments in the region and associated pressures on its water resources, including mine dewatering (abstraction) operations (Environmental Protection Authority 2018).

To address these uncertainties, we applied an integrative taxonomic framework combining multi‐locus molecular data and shell morphology to specimens of Velesunioninae collected across five drainage systems in north‐western Australia. Specifically, we aimed to (1) test whether the currently reported species from the region ( *V. wilsonii* , *V. angasi* and *L. froggatti* ) are supported by genetic evidence or instead comprise multiple lineages, and (2) evaluate the geographic distributions of the recovered lineages across drainage systems. We hypothesised that, consistent with patterns observed in other freshwater taxa from north‐western Australia, the apparently widespread Velesunioninae morpho‐species would comprise distinct genetic lineages with restricted drainage‐level distributions.

## 2 Methods

### 2.1 Sampling

Live Velesunioninae specimens (*n* = 43) were obtained between April 2021 and October 2025 from nine waterbodies from five drainages across north‐western Australia (Table 1). Specimens were collected via systematic and targeted hand foraging among soft (sand and silt) sediments and littoral bank margins, up to maximum wadable depths. Hand foraging was also undertaken in the hyporheic zone at Stony Creek, where saturated subsurface sediments were present under recessional wet season flows. Specimens were frozen whole for 24 h and preserved in 100% ethanol until analysis. All specimens were deposited with the Western Australian Museum (see Table A1 for registration numbers).

**TABLE 1:** Collection details for the Velesunioninae collected in this study.

Species	Site	Drainage	Date	Latitude	Longitude	*COI*	*16S*	*28S*	*18S*
*Lortiella froggatti*	Long Pool, Coongan River	De Grey River	15/05/2025	−20.9019	119.7886	12	5	5	4
	Lower Myroodah, Fitzroy River	Fitzroy River	5/10/2025	−18.0817	124.2198	2	2	1	
*Velesunio* ‘sp. Lineage A’	Rocky Waterhole, Sturt Creek	Sandy Desert	21/05/2025	−18.6659	128.5530	1	1	1	1
*Velesunio* ‘sp. Lineage E’	Stony Creek, Ord River	Ord‐Pentecost Rivers	25/05/2021	−17.3387	128.0497	9	9	8	4
	Dunham River Tributary	Ord‐Pentecost Rivers	15/06/2021	−16.4482	127.9584	1	1		
	Blina Creek, Fitzroy River	Fitzroy River	5/10/2025	−17.9624	124.2879	5	5	5	
*Velesunio* ‘sp. Lineage F’	Yule River	Port Hedland Coast	1/11/2021	−20.7015	118.2985	6	6	6	4
*Velesunio* ‘sp. Lineage G’	Anjammie Pool, Sturt Creek	Sandy Desert	23/05/2025	−18.7863	128.3233		1	1	1
	Rocky Waterhole, Sturt Creek	Sandy Desert	21/05/2025	−18.6659	128.5530	3	5	5	
	Stretch Lagoon, Sturt Creek	Sandy Desert	25/05/2025	−19.6791	127.5874	1	1	1	
Total						40	36	33	14

*Note:* Numbers in *COI*, *16S*, *28S* and *18S* columns refer to the number of individuals sequenced for that gene from each site. See Table A1 for GenBank accession and Western Australian Museum registration numbers for the material in this study. Drainage information was collected from Commonwealth of Australia (Bureau of Meteorology): https://www.bom.gov.au/water/about/riverBasinAuxNav.shtml.

### 2.2 DNA Extraction, Amplification and Sequencing

For each specimen, genomic DNA was extracted from approximately 2.5 mg of foot tissue, using the Qiagen DNeasy Blood and Tissue kit, following the manufacturer's instructions for tissue samples.

Polymerase chain reaction (PCR) was used to amplify four gene regions using published primer pairs (Table 2). Reaction mixtures consisted of 12.5 μL Bioline MyTaq RedMix, 1 μL (0.2 μM) of each primer, 9.5 μL of PCR grade water and 2 μL DNA.

**TABLE 2:** Primers used to amplify and sequence portions of *COI*, *16S, 28S* and *18S* in the Velesunioninae in this study.

Gene	Name	Direction	Sequence	Length (bp)	References
*COI*	LCO1490	F	5′‐GGTCAACAAATCATAAAGATATTGG‐3′	658	Folmer et al. (1994)
	HCO2198	R	5′‐TAAACTTCAGGGTGACCAAAAAATCA‐3′		
*16S*	16sar‐L	F	5′‐CGCCTGTTTATCAAAAACAT‐3′	~482	Palumbi et al. (2002)
	16sbr‐H	R	5′‐CCGGTCTGAACTCAGATCACGT‐3′		
*28S*	28sD1f	F	5′‐ACCCSCTGAAYTTAAGCAT‐3′	~1323	Colgan et al. (2003)
	28ffr	R	5′‐GGTGAGTTGTTACACACTCCTTAGCGGAT‐3′		Hillis and Dixon (1991)
*18S*	1F	F	5′‐TACCTGGTTGATCCTGCCAGTAG‐3′	~1668	Giribet et al. (1996)
	9R	R	5′‐GATCCTTCCGCAGGTTCACCTAC‐3′		

The PCR amplification conditions for the *COI* region were: (i) an initial denaturation phase of 5 min at 95°C; (ii) 7 cycles of 30 s denaturation at 95°C, 30 s of annealing at 45°C and 1 min of extension at 72°C then 35 cycles of 30 s denaturation at 95°C, 30 s of annealing at 50°C and 1 min of extension at 72°C; and (iii) a final 7 min extension at 72°C. The conditions for the *16S* region were: (i) an initial denaturation phase of 5 min at 95°C; (ii) 40 cycles of 30 s denaturation at 95°C, 30 s of annealing at 50°C and 1 min of extension at 72°C; and (iii) a final 7 min extension at 72°C. The *28S* and *18S* genes were amplified using: (i) an initial denaturation phase of 5 min at 95°C; (ii) 35 cycles of 45 s denaturation at 95°C, 1 min of annealing at 52°C and 1.5 min extension at 72°C; and (iii) a final 7 min extension at 72°C.

The outcomes of PCR assays were assessed using a 2% agarose gel stained with SYBR Safe (Invitrogen). Negative controls that contained no DNA were run in all PCR reactions to test for the presence of contaminants. PCR products were purified using Exo‐SAP (Dugan et al. 2002) and sequenced bidirectionally in an automatic ABI 3700 sequencer (Applied Biosystems) at Macrogen Inc. (Korea). The forward and reverse sequences were assembled into consensus sequences in Geneious Prime 2023 (Kearse et al. 2012). All 43 individuals were successfully sequenced for at least one genetic marker, although amplification success varied among loci (Table 1; Table A1).

### 2.3 Phylogenetic Analyses

The analysis comprised single locus *COI*, *16S* and *28S* datasets as well as a concatenated dataset of all genes. Although *18S* sequences were generated for 14 individuals including representatives of all lineages generated as part of this study, the data contained insufficient variation to be phylogenetically informative and were subsequently excluded from the analyses. The single gene analyses contained *COI* = 40, *16S* = 36 and *28S* = 33 individuals while the concatenated analysis comprised a total of 32 individuals successfully sequenced for all three genes. Additional *COI*, *16S* and *28S* sequences from the Velesunioninae, Hyriinae and Unionidae were downloaded from GenBank (Table A2).

All analysed genes were aligned using MAFFT plugin (Katoh et al. 2002) with the E‐INS‐I method and uncorrected genetic distances between haplotypes calculated for all markers. *Unio pictorum* (Unionidae) was used as the outgroup in all analyses following Graf et al. (2015). All generated sequences were uploaded to GenBank (see Table A1).

Phylogenetic relationships were inferred using maximum likelihood (ML) and Bayesian inference (BI) with the model of evolution selected using the corrected Akaike's information criterion in jModelTest v.2.1.10 (Darriba et al. 2012). ML analyses were conducted in Geneious Prime 2023 (Kearse et al. 2012) using the RAxML plugin under a GTR + G model with 1000 bootstrap replicates with nodes above 75% considered well supported (Lemey et al. 2009). Bayesian analyses were performed in MrBayes v.3.2.7 (Ronquist and Huelsenbeck 2003) using a GTR + G substitution model in a Markov chain Monte Carlo framework. Analyses were run for 10,000,000 generations with four concurrent chains (one cold and three heated; temperature = 0.2), sampling every 1000 generations. The first 25% of samples were discarded as burn‐in, and the remaining trees were used to estimate the majority‐rule consensus topology and Bayesian posterior probabilities (BPP). Only nodes with BPP > 0.90 were regarded as strongly supported (Drummond and Rambaut 2007). *COI* was partitioned by codon position, and concatenated analyses were partitioned into five subsets (*COI* codon positions 1–3, *16S*, *28S*).

### 2.4 Morphology

Notwithstanding the known uncertainties around identifying Velesunioninae taxa using the current morpho‐taxonomy, identification of specimens was done using McMichael and Hiscock (1958). Five shell measurements were recorded: total shell length (TL), beak height (BH), maximum shell height (MH), long beak length (LBL) and shell width (W) were measured as per McMichael and Hiscock (1958). A morphometric analysis was conducted on five shell ratios to test whether genetic lineages had characteristically different shell dimensions. These were the maximum height index (MHI = MH/TL), beak height index (BHI = BH/MH), beak length index (BLI = LBL/TL), width length index (WLI = W/TL), width height index (WHI = W/MH). Shell ratios were then analysed via Linear Discriminant Analysis (LDA) performed in R version 4.0.4. LDA was chosen because it models the difference between defined groups, here genetic lineages. All raw shell measurements and ratios are provided in Table A3.

## 3 Results

### 3.1 Phylogenetic Analysis

For clarity, the genetic lineages below have already been identified and assigned a name. Although larger fragments of *COI*, *16S* and *28S* were generated in this study, the analysed fragments (*COI* = 473 bp, *16S* = 415 bp and *28S* = 413 bp) were trimmed to match the lengths of the available comparable sequence data.

**FIGURE 1:**
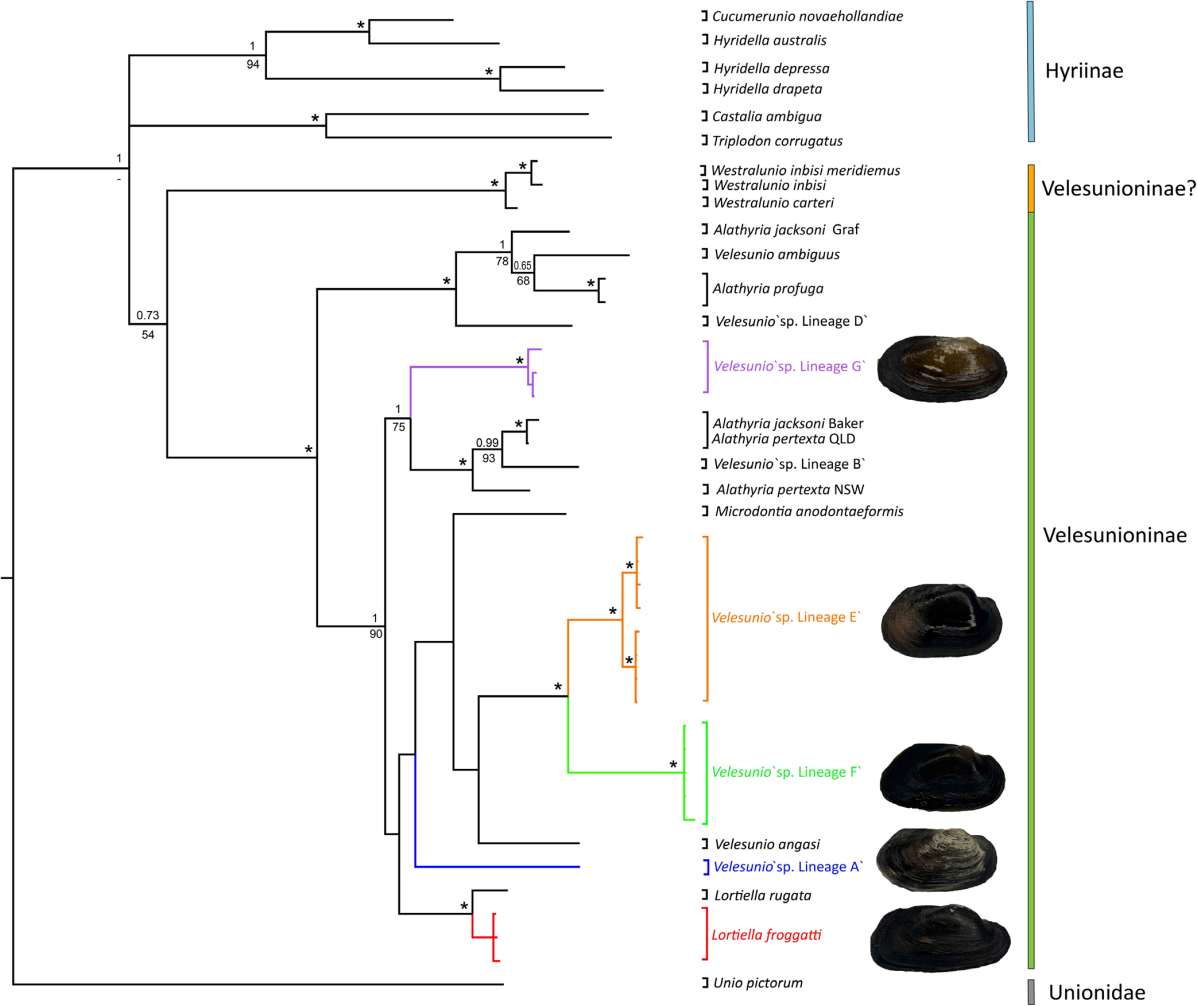
Phylogenetic tree depicting the relationship among Hyriidae lineages in the partitioned concatenated *COI*, *16S* and *28S* dataset (total of 1301 bp) estimated using Bayesian inference using the GTR + G model of evolution. Node support is indicated by Bayesian posterior probabilities (BPP; above) and bootstrap values (below) with * reflecting nodes with bootstrap support > 98% and BPP > 0.98. Lineages sequenced as part of this study have been coloured. Images of adjacent species are not to the same scale but are of sequenced specimens.

The concatenated dataset comprised a total of 1301 bp with 19 haplotypes generated in this study and 23 haplotypes downloaded from GenBank. The topologies of the phylogenetic trees inferred by ML and BI analyses of this dataset were essentially congruent although the degree of support for some branching patterns did differ (Figure 1; see Appendix A for single gene trees).

**FIGURE 2:**
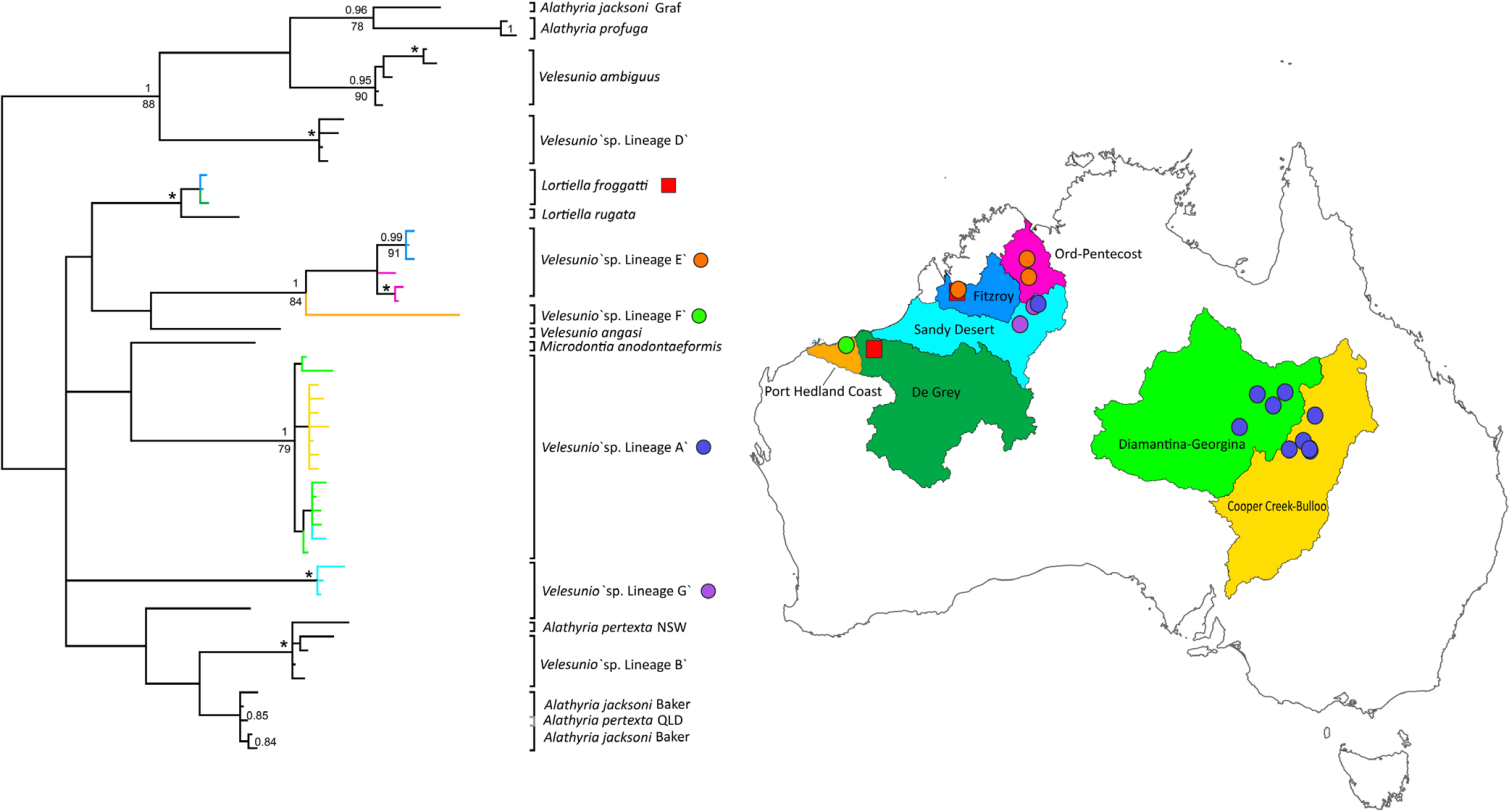
*COI* phylogenetic tree depicting the relationships among the Velesunioninae (excluding *Westralunio*) estimated using Bayesian inference and the GTR + G model of evolution. Node support is indicated by Bayesian posterior probabilities (BPP; above) and bootstrap values (below) with * reflecting nodes with bootstrap support > 98% and BPP > 0.98.

**FIGURE A1:**
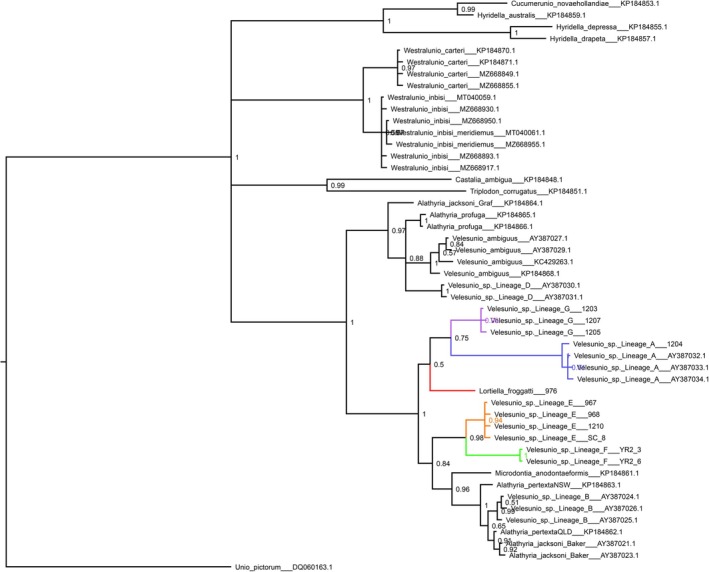
Phylogenetic tree inferred using Bayesian approaches for the *16S* dataset. Node support values are Bayesian posterior probabilities converted to percentages. Only nodes with BPP values > 0.95 (95) have been annotated. Lineages sequenced as part of this study are coloured.

**FIGURE A2:**
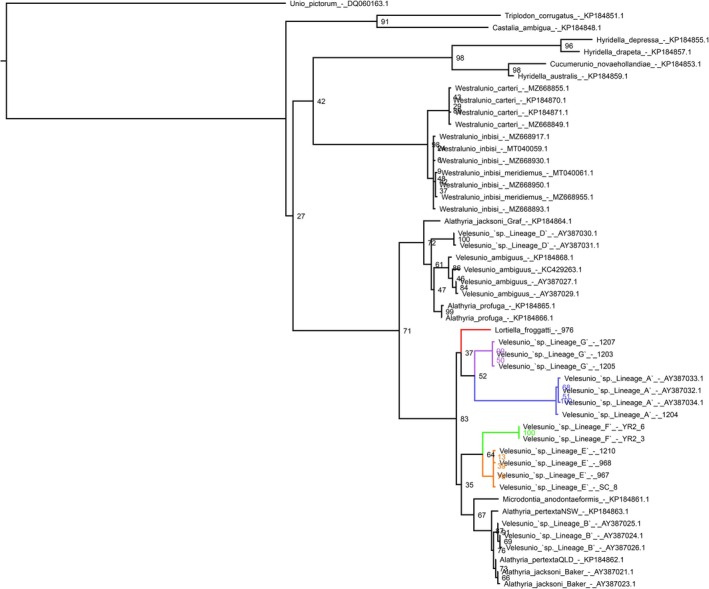
Phylogenetic tree inferred using maximum likelihood for the *16S* dataset. Node support values are bootstrap values. Only nodes with > 75% bootstrap support have been annotated. Lineages sequenced as part of this study are coloured.

**FIGURE A3:**
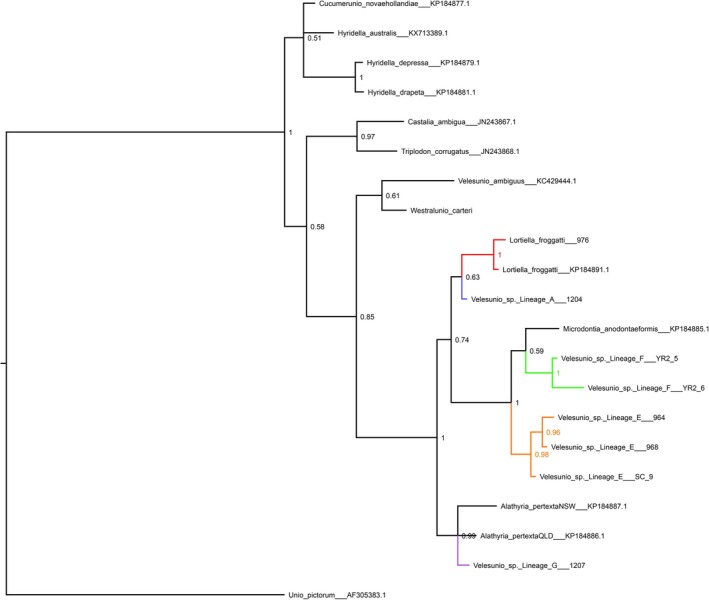
Phylogenetic tree inferred using Bayesian inference for the *28S* dataset. Node support values are Bayesian posterior probabilities (BPP) converted to percentages. Only nodes with BPP values > 0.95 (95) have been annotated. Lineages sequenced as part of this study are coloured.

**FIGURE A4:**
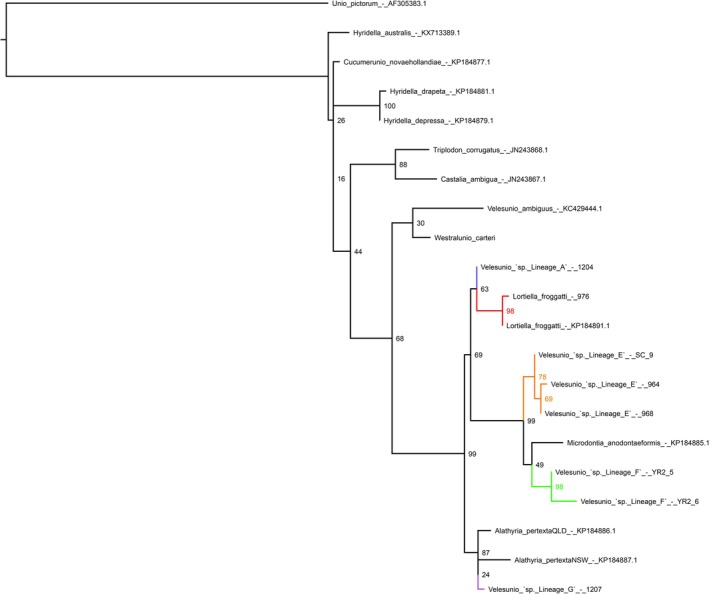
Phylogenetic tree inferred using maximum likelihood for the *28S* dataset. Node support values are bootstrap values. Only nodes with > 75% bootstrap support have been annotated. Lineages sequenced as part of this study are coloured.

Phylogenetic analyses of the concatenated dataset resolved the Hyriinae and Velesunioninae as distinct clades, although the support separating the clades received strong support in the BI analysis (BPP = 1) but not in the ML analysis. The position of the *Westralunio* lineages within the Velesunioninae was not recorded with statistically significant support in the concatenated (Figure 1), *COI* (Figure 2), *16S* or *28S* trees (Figures A1, A2, A3, A4). However, within the Velesunioninae, *Velesunio*, *Alathyria*, *Microdontia* and *Lortiella* formed a universally well‐supported clade (Figure 1). As expected, neither *Velesunio* nor *Alathyria* lineages formed reciprocally monophyletic clades in any of the analyses (e.g., Figure 1). The two *Lortiella* lineages ( *L. froggatti* and *L. rugata* ) did form a well‐supported clade (BPP = 1, BS = 100%).

### 3.2 Species Delimitation

The specimens sequenced in this study formed five monophyletic lineages in the ML and BI analysis of all genes. Across the three loci, maximum intraspecific divergences within these lineages were consistently lower than the minimum interspecific divergences between lineages (Table 3). *COI* maximum intraspecific distances for these five lineages were 2.3%, whereas minimum interspecific distances were 2.5%. Similarly, *16S* exhibited low intraspecific variation (maximum = 0.8%) relative to interspecific divergence (minimum = 4.5%), while *28S* showed very limited intraspecific divergences but these were still lower than the minimum interspecific distances between each lineage (Table 3).

**TABLE 3:** Summary of intraspecific (Intra) and interspecific (Inter) uncorrected pairwise distances for *COI*, *16S* and *28S* generated during this study.

Species	*COI*		*16S*		*28S*	
	Intra	Inter	Intra	Inter	Intra	Inter
*Lortiella froggatti*	0.21–0.42	2.54–17.12	NA	5.54–22.42	0.26	1.03–19.33
*Velesunio* ‘sp. Lineage A’	0.21–2.33	7.82–18.60	0.25–0.76	7.56–23.99	NA	1.03–18.39
*Velesunio* ‘sp. Lineage E’	0.21–2.11	7.19–18.18	0.25–0.50	4.52–22.11	0.25–0.51	1.53–19.74
*Velesunio* ‘sp. Lineage F’	NA	7.19–18.05	0.13	4.52–21.97	1.02	1.53–21.13
*Velesunio* ‘sp. Lineage G’	0.21–1.06	9.51–18.60	0.25–0.50	5.28–23.17	NA	0.77–18.39

*Note:* Pairwise distances are presented as percentages in the format minimum–maximum. NA values indicate the taxon was represented by a single haplotype.

The *COI* and *16S* sequences of ‘ *Alathyria jacksoni* ’ reported by Graf et al. (2015) and Baker et al. (2004) differ by 10.7% and 9.8% respectively, and were resolved as distinct lineages in the phylogenetic analysis (e.g., Figures 1 and 2). In addition, one of the two ‘ *Alathyria pertexta* ’ specimens reported in Graf et al. (2015) from Queensland, Australia had *COI* and *16S* pairwise differences of 0.21% and 0.25% from the ‘ *Alathyria jacksoni* ’ sequences reported by Baker et al. (2004). The second ‘ *Alathyria pertexta* ’ specimen from New South Wales, Australia formed a distinct branch which was at least 5.7% (*COI*) and 1.6% (*16S*) divergent from the next most similar sequence in the analysis (see Table A4).

### 3.3 Drainage Patterns

Two of the five lineages (*V*. ‘sp. Lineage F’ and *V*. ‘sp. Lineage G’) were collected from one drainage while the remaining three lineages were found across two (*V*. ‘sp. Lineage E’ and *L. froggatti* ) or three (*V*. ‘sp. Lineage A’) drainages, respectively. *Velesunio* ‘sp. Lineage A’ displayed genetic structuring corresponding with two drainages (Diamantina‐Georgina and Cooper Creek‐Bulloo) in central‐eastern Australia with the specimen collected from the Sandy Desert nested within haplotypes from Diamantina‐Georgina (Figure 2). Despite a geographic distance of 1320 km, the minimum pairwise *COI* divergence between these drainages was 0.42%. Similarly, *L. froggatti* collected from the Fitzroy and De Grey drainage areas, which are separated by 550 km, had minimum pairwise *COI* divergences of 0.21% while the two *V*. ‘sp. Lineage E’ populations collected from the Ord‐Pentecost differed more from each other (1.69%) than to the Fitzroy population (1.26%).

### 3.4 Morphology

The shells of four lineages: *V*. ‘sp. Lineage A’, *V*. ‘sp. Lineage E’, *V*. ‘sp. Lineage F’ and *V*. ‘sp. Lineage G’ were identified as *Velesunio* while one lineage ( *L. froggatti* ) was identified as *Lortiella*. *Velesunio* ‘sp. Lineage E’ and *V*. ‘sp. Lineage F’ resembled the morphological description of *V. angasi* , while *V*. ‘sp. Lineage A’ and *V*. ‘sp. Lineage G’ displayed shell morphologies consistent with *V. wilsonii* . The morphologies of the *Lortiella* specimens were consistent with those of *L. froggatti* (Table A3).

**FIGURE 3:**
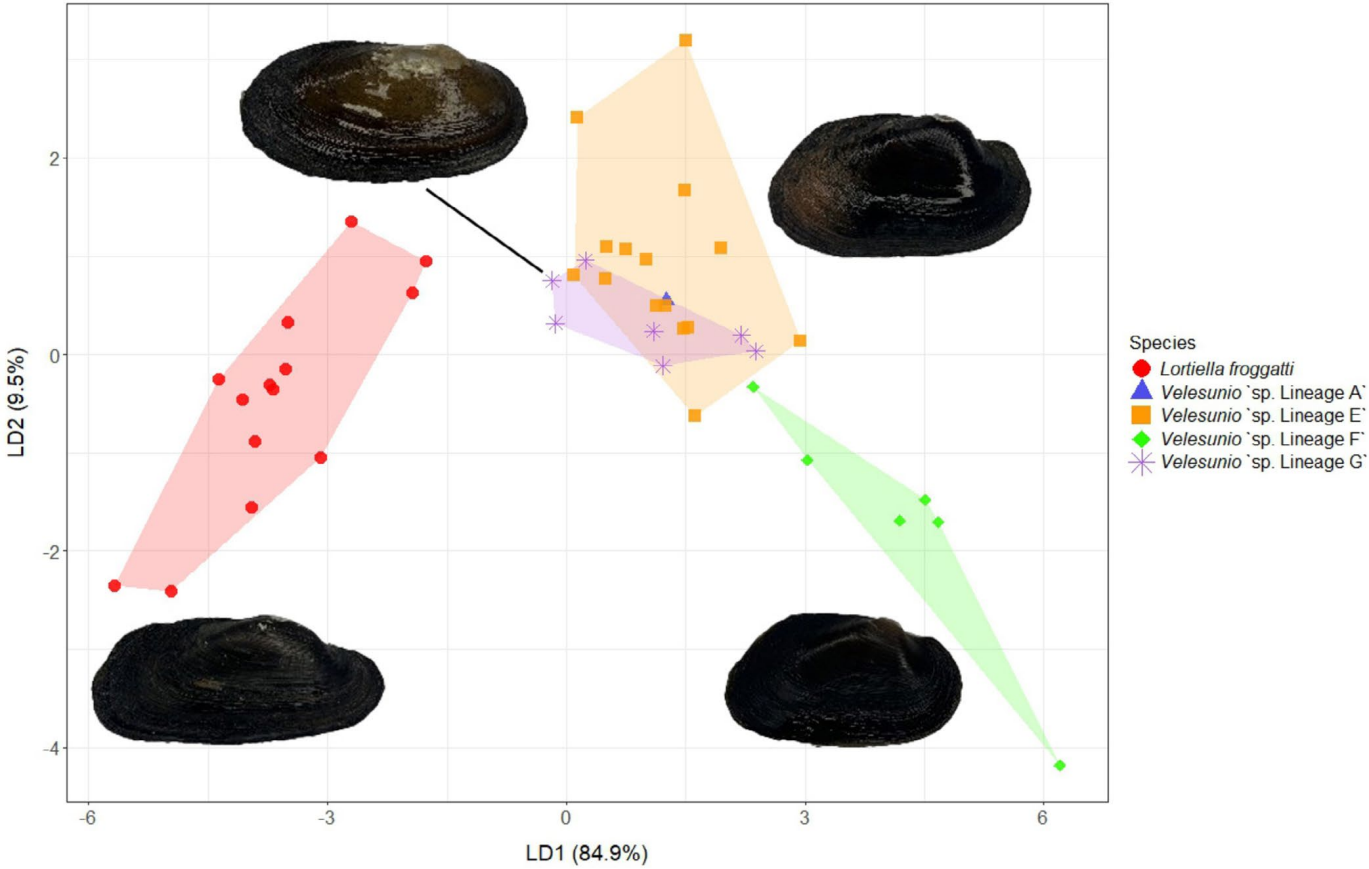
Linear discriminant analysis (LDA) of shell morphometric ratios for the Velesunioninae species included in this study. Points represent individual specimens, coloured and shaped by lineage with shaded polygons indicating convex hulls encompassing each group.

All 43 sequenced specimens were included in the morphometric analysis. Lineage separation was dominated by the first discriminant axis (LD1 = 84.9%), with only minor additional separation along the second axis (LD2 = 9.5%; Figure 3). Leave‐one‐out cross‐validated LDA correctly classified all individuals of *L. froggatti* , 83.3% of *V.* ‘sp. Lineage F’, 80% of *V.* ‘sp. Lineage E’ and 71.4% of *V.* ‘sp. Lineage G’. Misclassification occurred primarily between *V.* ‘sp. Lineage E’ and *V.* ‘sp. Lineage G’. *V.* ‘sp. Lineage A’ clustered within the morphospace of *V.* ‘sp. Lineage E’ and *V.* ‘sp. Lineage G’, but the single specimen was excluded from the leave‐one‐out cross‐validation analysis.

Separation along LD1 was driven primarily by the maximum height index which separated the narrowly elongated shells of *L. froggatti* from the broader shelled *Velesunio* lineages. *Velesunio* ‘sp. Lineage F’ exhibited the most distinct shell shapes of the four *Velesunio* lineages, separated primarily by relatively wider, more laterally inflated shells and lower shell height relative to length.

## 4 Discussion

Analyses of multiple molecular loci revealed five distinct Velesunioninae lineages, indicating that the biodiversity of this group in north‐western Australia has been underestimated and that the distributions of some species are likely more restricted than previously assumed. Except for *L. froggatti* which displayed a distinct shell morphology, morphological characters were generally insufficient to reliably distinguish among the lineages, highlighting the importance of molecular data for resolving species boundaries in this group. Our phylogenetic analyses also indicate that the position of *Westralunio* within the Hyriidae requires further clarification. Together, these findings have important implications for understanding the diversity, taxonomy and management of freshwater mussels in north‐western Australia.

### 4.1 Velesunioninae in North‐Western Australia

The specimens collected in this study were morphologically consistent with the three Velesunioninae species currently reported from north‐western Australia ( *L. froggatti* , *V. wilsonii* and *V. angasi* ). However, *L. froggatti* was the only species that could be confidently identified from its shell morphology. In contrast, both *V. wilsonii* and *V. angasi* morphotypes contained multiple highly divergent genetic lineages. Specimens assigned morphologically to *V. wilsonii* comprised *V.* ‘sp. Lineage A’ and *V.* ‘sp. Lineage G’, with the former lineage previously identified by Baker et al. (2003) as one of three genetically distinct eastern Australian lineages exhibiting this morphotype. Similarly, specimens resembling *V. angasi* comprised two deeply divergent lineages (*V.* ‘sp. Lineage E’ and *V.* ‘sp. Lineage F’), both of which differed from the published *COI* sequence for *V. angasi* generated by Bogan and Hoeh (2000), assuming the identity of this sequence has been correctly assigned. These findings suggest that the apparent broad distributions previously attributed to *V. wilsonii* and *V. angasi* may instead reflect complexes of morphologically similar species with more restricted geographic ranges (although see *V*. ‘sp. Lineage A’ below). It is also possible that *V. wilsonii* and *V. angasi sensu stricto* do occur in north‐western Australia, however, until the taxonomy of these species is clarified, their currently reported distributions should be treated with caution.

This pattern of previously unrecognised diversity is consistent with broader phylogeographic patterns observed in freshwater taxa across north‐western Australia, where molecular studies have revealed strong genetic structuring and limited connectivity among populations that were previously assumed to be widely distributed (Huey et al. 2014). Because freshwater mussels disperse to new habitats primarily via parasitic attachment of their glochidia to host fishes (Daviot et al. 2026; Klunzinger et al. 2010, 2012), patterns of distribution and genetic structure are ultimately shaped by the movement and connectivity of their hosts. In eastern Australia, population genetic studies of *Velesunio* including *V*. ‘sp. Lineage A’ have suggested that contemporary gene flow is often strongly restricted both between drainages and even among waterholes within the same drainage, implying limited effective dispersal via host fishes (Hughes et al. 2004). Interestingly, we observed very little differentiation at *COI* between the population of *Velesunio* ‘sp. Lineage A’ collected here from its previously known distribution in central‐eastern Australia approximately 1320 km away. Similarly, the two *L. froggatti* populations examined here showed low levels of *COI* divergence despite occurring in different drainage systems currently separated by the Great Sandy Desert. These patterns suggest that rare long‐distance dispersal events may occasionally occur, potentially facilitated by highly mobile and widely distributed host fishes (e.g., spangled perch *Leiopotherapon unicolor* ; Bostock et al. 2006) during major flood events, or that connectivity among drainage systems was greater under past climatic regimes and sea‐level conditions than is observed today. However, disentangling the relative contributions of these factors to the distributional patterns observed among lineages remains difficult from the current sampling. Resolving this will require broader population‐level sampling across drainages and an assessment of host associations and habitat conditions.

### 4.2 Velesunioninae

Our phylogenetic analyses recovered *Westralunio* as a sister lineage to a well‐supported clade comprising the remaining Velesunioninae genera (*Velesunio*, *Alathyria*, *Lortiella* and *Microdontia*), although this relationship was not significantly supported in either the concatenated or single‐gene analyses. Similar results have been reported in previous molecular phylogenies of the Hyriidae, where *Westralunio* was recovered outside a well‐supported clade containing *Velesunio*, *Alathyria*, *Lortiella* and *Microdontia*, with no alternative placement receiving strong statistical support (da Cruz Santos‐Neto et al. 2016; Graf et al. 2015). The Velesunioninae was originally erected for Australian genera characterised by smooth umbos (Iredale 1934), although this diagnostic character has subsequently been shown to be inconsistent across the group (Zieritz et al. 2013). The unstable placement of *Westralunio* in phylogenetic analyses is unclear but may reflect incomplete taxon sampling within the Velesunioninae, particularly given the discovery of multiple novel lineages in this study and in earlier work by Baker et al. (2003). Future studies incorporating broader taxon sampling and genome‐scale phylogenomic data will likely be necessary to fully resolve the evolutionary relationships and composition of the Velesunioninae.

### 4.3 Conservation Implications

These results highlight the need for further targeted sampling of freshwater mussels in north‐western Australia. It should be a conservation priority to better document Velesunioninae diversity, formally describe undescribed lineages, and assess species distributions, given that some waterbodies in the region are affected by resource extraction that may be highly detrimental to the fauna of affected ecosystems (Environmental Protection Authority 2018). Genetic data are especially critical to this end given (1) the possibility of species co‐occurrence which is not uncommon in eastern Australia (Baker et al. 2003, 2004) and also was documented for *V*. ‘sp. Lineage A’ and *V*. ‘sp. Lineage G’ at one site in this study (Rocky Waterhole), and (2) the unreliability of morphological data for species identification. Given effective conservation management is contingent on identifying species (Tsang et al. 2016), the unresolved taxonomy of the Velesunioninae remains a major barrier in assessing their conservation status (Walker et al. 2014).

Developing targeted non‐invasive genetic methods that can reliably distinguish Australian freshwater mussels would be beneficial for establishing a basic understanding of the presence and distribution of different species without destructive sampling. This could include targeted environmental DNA (eDNA) surveys or minimally invasive swabbing methods used for other bivalves of conservation concern (Harrison et al. 2023). eDNA may also assist in surveying for freshwater mussels where conventional survey methods (e.g., hand foraging on littoral banks) are complicated by poor water clarity and the presence of fresh and/or estuarine crocodile populations.

## 5 Conclusions

Using a multi‐locus phylogenetic analysis, we identified five lineages from five drainages across north‐western Australia, including *L. froggatti* , previously recognised *V*. ‘sp. Lineage A’ and three undescribed *Velesunio* lineages. Morphometric data reinforced that shell morphology can be used to separate *L. froggatti* from *Velesunio* species but is less effective at discriminating among *Velesunio* lineages. Collectively, our findings indicate that the Velesunioninae diversity in north‐western Australia has been underestimated and targeted surveys that utilise genetic data are required to better resolve species distributions and to test for additional, regionally endemic diversity. Further work is crucial to develop robust conservation assessments in a region where freshwater ecosystems are increasingly at risk from resource development.

## Author Contributions

**Angus D' Arcy Lawrie:** data curation (equal), formal analysis (equal), investigation (equal), project administration (equal), writing – original draft (equal), writing – review and editing (equal). **Jake Ryan Daviot:** data curation (equal), methodology (equal), resources (equal), writing – original draft (equal), writing – review and editing (equal). **Christopher Hofmeester:** data curation (equal), investigation (equal), resources (equal), writing – original draft (equal), writing – review and editing (equal). **Adam Harman:** conceptualization (equal), data curation (equal), investigation (equal), project administration (equal), resources (equal), writing – original draft (equal), writing – review and editing (equal). **Joel Huey:** formal analysis (equal), supervision (equal), writing – original draft (equal), writing – review and editing (equal).

## Funding

This work was supported by Fortescue, Northern Minerals and Panoramic Resources.

## Conflicts of Interest

The authors declare no conflicts of interest.

## Data Availability

Data generated in this study can be found in the Appendix A and also on GenBank (see text for accession numbers). All specimens have been submitted to the Western Australian Museum.

## Appendix A

**TABLE A1:** List of all specimens sequenced in this study and their associated Western Australian Museum (WAM) registration and GenBank accession numbers.

Individual code	WAM reg	Site	Sampling date	Latitude	Longitude	Species	*16S*	*28S*	*COI*	*18S*
864	WAM97279	Coongan River	15/05/2025	−20.9019	119.7886	*Lortiella froggatti*	PX737783	PX737819	PX734090	
865	WAM97280	Coongan River	15/05/2025	−20.9019	119.7886	*Lortiella froggatti*	PX737784	PX737820	PX734091	PX737852
866	WAM97281	Coongan River	15/05/2025	−20.9019	119.7886	*Lortiella froggatti*	PX737785	PX737821	PX734092	PX737853
867	WAM97282	Coongan River	15/05/2025	−20.9019	119.7886	*Lortiella froggatti*	PX737786	PX737822	PX734093	PX737854
868	WAM97283	Coongan River	15/05/2025	−20.9019	119.7886	*Lortiella froggatti*	PX737787	PX737823	PX734094	PX737855
869	WAM97284	Coongan River	15/05/2025	−20.9019	119.7886	*Lortiella froggatti*			PX734095	
871	WAM97286	Coongan River	15/05/2025	−20.9019	119.7886	*Lortiella froggatti*			PX734096	
872	WAM97287	Coongan River	15/05/2025	−20.9019	119.7886	*Lortiella froggatti*			PX734097	
873	WAM97288	Coongan River	15/05/2025	−20.9019	119.7886	*Lortiella froggatti*			PX734098	
874	WAM97289	Coongan River	15/05/2025	−20.9019	119.7886	*Lortiella froggatti*			PX734099	
875	WAM97290	Coongan River	15/05/2025	−20.9019	119.7886	*Lortiella froggatti*			PX734100	
889	WAM97291	Coongan River	15/05/2025	−20.9019	119.7886	*Lortiella froggatti*			PX734101	
975	WAM97297	Lower Myroodah	5/10/2025	−18.0817	124.2198	*Lortiella froggatti*	PX737793		PX734102	
976	WAM97298	Lower Myroodah	5/10/2025	−18.0817	124.2198	*Lortiella froggatti*	PX737794	PX737829	PX734103	
1204	WAM97303	Rocky Waterhole	21/05/2025	−18.6659	128.5531	*Velesunio* ‘sp. Lineage A’	PX737799	PX737834	PX734131	PX737857
964	WAM97292	Blina Creek Flood Plain	5/10/2025	−17.9624	124.288	*Velesunio* ‘sp. Lineage E’	PX737788	PX737824	PX734110	
965	WAM97293	Blina Creek Flood Plain	5/10/2025	−17.9624	124.288	*Velesunio* ‘sp. Lineage E’	PX737789	PX737825	PX734111	
966	WAM97294	Blina Creek Flood Plain	5/10/2025	−17.9624	124.288	*Velesunio* ‘sp. Lineage E’	PX737790	PX737826	PX734112	
967	WAM97295	Blina Creek Flood Plain	5/10/2025	−17.9624	124.288	*Velesunio* ‘sp. Lineage E’	PX737791	PX737827	PX734113	
968	WAM97296	Blina Creek Flood Plain	5/10/2025	−17.9624	124.288	*Velesunio* ‘sp. Lineage E’	PX737792	PX737828	PX734114	
1210	WAM97307	DRTS1	15/06/2021	−16.4482	127.9584	*Velesunio* ‘sp. Lineage E’	PX737803		PX734115	
SC_1	WAM97308	Stony Creek	25/05/2021	−17.3387	128.0497	*Velesunio* ‘sp. Lineage E’	PX737804	PX737838	PX734116	
SC_2	WAM97309	Stony Creek	25/05/2021	−17.3387	128.0497	*Velesunio* ‘sp. Lineage E’	PX737805	PX737839	PX734119	PX737858
SC_3	WAM97310	Stony Creek	25/05/2021	−17.3387	128.0497	*Velesunio* ‘sp. Lineage E’	PX737806		PX734120	
SC_4	WAM97311	Stony Creek	25/05/2021	−17.3387	128.0497	*Velesunio* ‘sp. Lineage E’	PX737807	PX737840	PX734121	PX737859
SC_5	WAM97312	Stony Creek	25/05/2021	−17.3387	128.0497	*Velesunio* ‘sp. Lineage E’	PX737808	PX737841	PX734122	
SC_6	WAM97313	Stony Creek	25/05/2021	−17.3387	128.0497	*Velesunio* ‘sp. Lineage E’	PX737809	PX737842	PX734117	PX737860
SC_7	WAM97314	Stony Creek	25/05/2021	−17.3387	128.0497	*Velesunio* ‘sp. Lineage E’	PX737810	PX737843	PX734118	
SC_8	WAM97315	Stony Creek	25/05/2021	−17.3387	128.0497	*Velesunio* ‘sp. Lineage E’	PX737811	PX737844	PX734123	PX737861
SC_9	WAM97316	Stony Creek	25/05/2021	−17.3387	128.0497	*Velesunio* ‘sp. Lineage E’	PX737812	PX737845	PX734124	
YR2_1	WAM97317	Yule River	1/11/2021	−20.7016	118.2986	*Velesunio* ‘sp. Lineage F’	PX737813	PX737846	PX734125	PX737862
YR2_2	WAM97318	Yule River	1/11/2021	−20.7016	118.2986	*Velesunio* ‘sp. Lineage F’	PX737814	PX737847	PX734126	
YR2_3	WAM97319	Yule River	1/11/2021	−20.7016	118.2986	*Velesunio* ‘sp. Lineage F’	PX737815	PX737848	PX734127	PX737863
YR2_4	WAM97320	Yule River	1/11/2021	−20.7016	118.2986	*Velesunio* ‘sp. Lineage F’	PX737816	PX737849	PX734129	PX737864
YR2_5	WAM97321	Yule River	1/11/2021	−20.7016	118.2986	*Velesunio* ‘sp. Lineage F’	PX737817	PX737850	PX734130	
YR2_6	WAM97322	Yule River	1/11/2021	−20.7016	118.2986	*Velesunio* ‘sp. Lineage F’	PX737818	PX737851	PX734128	PX737865
1200	WAM97299	Anjammie Pool	23/05/2025	−18.7864	128.3233	*Velesunio* ‘sp. Lineage G’	PX737795	PX737830	PX734104	PX737856
1201	WAM97300	Stretch Lagoon	25/05/2025	−19.6791	127.5875	*Velesunio* ‘sp. Lineage G’	PX737796	PX737831	PX734107	
1202	WAM97301	Rocky Waterhole	21/05/2025	−18.6659	128.5531	*Velesunio* ‘sp. Lineage G’	PX737797	PX737832		
1203	WAM97302	Rocky Waterhole	21/05/2025	−18.6659	128.5531	*Velesunio* ‘sp. Lineage G’	PX737798	PX737833	PX734105	
1205	WAM97304	Rocky Waterhole	21/05/2025	−18.6659	128.5531	*Velesunio* ‘sp. Lineage G’	PX737800	PX737835	PX734109	
1206	WAM97305	Rocky Waterhole	21/05/2025	−18.6659	128.5531	*Velesunio* ‘sp. Lineage G’	PX737801	PX737836	PX734106	
1207	WAM97306	Rocky Waterhole	21/05/2025	−18.6659	128.5531	*Velesunio* ‘sp. Lineage G’	PX737802	PX737837	PX734108	

**TABLE A2:** List of taxa downloaded from GenBank with associated accession numbers.

Species	Family	Subfamily	*COI*	*16S*	*28S*
*Castalia ambigua*	Hyriidae	Hyriinae	JN243889.1	KP184848.1	JN243867.1
*Cucumerunio novaehollandiae*	Hyriidae	Hyriinae	KP184901.1	KP184853.1	KP184877.1
*Hyridella australis*	Hyriidae	Hyriinae	AF305367.1	KP184859.1	KX713389.1
*Hyridella depressa*	Hyriidae	Hyriinae	KP184903.1	KP184855.1	KP184879.1
*Hyridella drapeta*	Hyriidae	Hyriinae	KP184905.1	KP184857.1	KP184881.1
*Triplodon corrugatus*	Hyriidae	Hyriinae	JN243890.1	KP184851.1	JN243868.1
*Alathyria jacksoni* Baker	Hyriidae	Velesunioninae	AY386975.1; AY386976.1; AY386977.1; AY386981.1	AY387021.1; AY387023.1	
*Alathyria jacksoni* Graf	Hyriidae	Velesunioninae	KP184912.1	KP184864.1	
*Alathyria pertexta* NSW	Hyriidae	Velesunioninae	KP184911.1	KP184863.1	KP184887.1
*Alathyria pertexta* QLD	Hyriidae	Velesunioninae	KP184910.1	KP184862.1	KP184886.1
*Alathyria profuga*	Hyriidae	Velesunioninae	KP184913.1; KP184914.1	KP184865.1; KP184866.1	
*Lortiella froggatti*	Hyriidae	Velesunioninae		KP184867.1	KP184891.1
*Lortiella rugata*	Hyriidae	Velesunioninae	AF231746.1		
*Microdontia anodontaeformis*	Hyriidae	Velesunioninae	KP184909.1	KP184861.1	KP184885.1
*Velesunio* ‘sp. Lineage A’	Hyriidae	Velesunioninae	AY211550.1; AY211551.1; AY211552.1; AY211553.1; AY211554.1; AY211555.1; AY211556.1; AY386982.1; AY386983.1; AY386984.1; AY386985.1; AY386986.1; AY386987.1; AY386988.1	AY387032.1; AY387033.1; AY387034.1	
*Velesunio* ‘sp. Lineage B’	Hyriidae	Velesunioninae	AY211557.1; AY211558.1; AY211559.1; AY211560.1; AY211561.1	AY387024.1; AY387025.1; AY387026.1	
*Velesunio* ‘sp. Lineage D’	Hyriidae	Velesunioninae	AY211587.1; AY211588.1; AY211589.1; AY211590.1	AY387030.1; AY387031.1	
*Velesunio ambiguus*	Hyriidae	Velesunioninae	AY211570.1; AY211571.1; AY211572.1; AY211573.1; AY211574.1	AY387027.1; AY387029.1; KC429263.1; KP184868.1	KC429444.1
*Velesunio angasi*	Hyriidae	Velesunioninae	AF231743.1		
*Westralunio carteri*	Hyriidae	Velesunioninae	KP184917.1; MT040629.1; MT040650.1; MT040651.1	KP184870.1; KP184871.1; MZ668849.1; MZ668855.1	MZ669121.1
*Westralunio inbisi*	Hyriidae	Velesunioninae	MT040633.1; MT040636.1; MT040659.1; MT040661.1; MT040662.1	MT040059.1; MZ668893.1; MZ668917.1; MZ668930.1; MZ668950.1	
*Westralunio inbisi meridiemus*	Hyriidae	Velesunioninae	MT040641.1; MZ668761.1; MZ668811.1; MZ668835.1	MT040061.1; MZ668955.1	
*Unio pictorum*	Unionidae		AF156499.1	DQ060163.1	AF305383.1

*Note:* Species names with additional identifiers correspond to sequences from: Baker = Baker et al. (2004), Graf = Graf et al. (2015), NSW/QLD = Graf et al. (2015).

**TABLE A3:** Shell measurements for individuals analysed in this study.

Individual code	WAM reg	Site	Species	TL	BH	MH	LBL	W	MHI	BHI	BLI	WLI	WHI
864	WAM97279	Coongan River	*Lortiella froggatti*	56.5	24.9	25.8	32.7	22.2	0.46	0.97	0.58	0.39	0.86
865	WAM97280	Coongan River	*Lortiella froggatti*	67.2	31	32.9	44.3	26	0.49	0.94	0.66	0.39	0.79
866	WAM97281	Coongan River	*Lortiella froggatti*	59.5	24.4	25.4	39.5	21	0.43	0.96	0.66	0.35	0.83
867	WAM97282	Coongan River	*Lortiella froggatti*	52.2	23	24.3	33.4	19.7	0.47	0.95	0.64	0.38	0.81
868	WAM97283	Coongan River	*Lortiella froggatti*	54.9	21.6	23.4	33.9	20.6	0.43	0.92	0.62	0.38	0.88
869	WAM97284	Coongan River	*Lortiella froggatti*	69.3	31.8	33.9	45.4	24.6	0.49	0.94	0.66	0.35	0.73
871	WAM97286	Coongan River	*Lortiella froggatti*	56.9	25.2	27.2	38.8	19.5	0.48	0.93	0.68	0.34	0.72
872	WAM97287	Coongan River	*Lortiella froggatti*	72.2	30.3	32.6	48.1	26	0.45	0.93	0.67	0.36	0.8
873	WAM97288	Coongan River	*Lortiella froggatti*	81.9	35.2	35.5	51.2	29.1	0.43	0.98	0.63	0.36	0.82
874	WAM97289	Coongan River	*Lortiella froggatti*	71.4	30.2	31.5	45.7	26.1	0.44	0.96	0.64	0.37	0.83
875	WAM97290	Coongan River	*Lortiella froggatti*	84.5	34.6	35.8	51.3	31.7	0.42	0.97	0.61	0.38	0.89
889	WAM97291	Coongan River	*Lortiella froggatti*	80.6	34	36.3	47.8	28	0.45	0.94	0.59	0.35	0.77
975	WAM97297	Lower Myroodah	*Lortiella froggatti*	75.4	27.8	34.2	50	21.3	0.45	0.81	0.66	0.28	0.62
976	WAM97298	Lower Myroodah	*Lortiella froggatti*	81.2	30	35.4	54.9	24.2	0.44	0.85	0.68	0.30	0.68
1204	WAM97303	Rocky Waterhole	*Velesunio* ‘sp. Lineage A’	82.5	41.5	43.6	54.6	27.4	0.53	0.95	0.66	0.33	0.63
964	WAM97292	Blina Creek Flood Plain	*Velesunio* ‘sp. Lineage E’	68.8	36.4	37.4	44	27	0.54	0.97	0.64	0.39	0.72
965	WAM97293	Blina Creek Flood Plain	*Velesunio* ‘sp. Lineage E’	27.8	16.4	16.4	15.6	11.65	0.59	1.00	0.56	0.42	0.71
966	WAM97294	Blina Creek Flood Plain	*Velesunio* ‘sp. Lineage E’	43.6	23.8	25.7	28.1	16.6	0.59	0.93	0.64	0.38	0.65
967	WAM97295	Blina Creek Flood Plain	*Velesunio* ‘sp. Lineage E’	43.7	24.1	26.2	26.8	17.4	0.60	0.92	0.61	0.40	0.66
968	WAM97296	Blina Creek Flood Plain	*Velesunio* ‘sp. Lineage E’	49	27.5	28.3	28.8	17.3	0.58	0.97	0.59	0.35	0.61
1210	WAM97307	DRTS1	*Velesunio* ‘sp. Lineage E’	43	22	23	28	15	0.53	0.96	0.65	0.35	0.65
SC_1	WAM97308	Stony Creek	*Velesunio* ‘sp. Lineage E’	59.5	30	31.6	38.5	21	0.53	0.95	0.65	0.35	0.66
SC_2	WAM97309	Stony Creek	*Velesunio* ‘sp. Lineage E’	59	28.1	31.8	42.4	20.7	0.54	0.88	0.72	0.35	0.65
SC_3	WAM97310	Stony Creek	*Velesunio* ‘sp. Lineage E’	53.3	25.5	29.9	40	17.5	0.56	0.85	0.75	0.33	0.59
SC_4	WAM97311	Stony Creek	*Velesunio* ‘sp. Lineage E’	57.3	28.7	30.3	39.6	20	0.53	0.95	0.69	0.35	0.66
SC_5	WAM97312	Stony Creek	*Velesunio* ‘sp. Lineage E’	47	23.1	23.6	29.9	15.4	0.50	0.98	0.64	0.33	0.65
SC_6	WAM97313	Stony Creek	*Velesunio* ‘sp. Lineage E’	54.3	27.1	28.4	35.6	17.3	0.52	0.95	0.66	0.32	0.61
SC_7	WAM97314	Stony Creek	*Velesunio* ‘sp. Lineage E’	58.5	29.5	30.7	37.3	19.2	0.52	0.96	0.64	0.33	0.63
SC_8	WAM97315	Stony Creek	*Velesunio* ‘sp. Lineage E’	59.1	29.3	31	39.8	19.4	0.52	0.95	0.67	0.33	0.63
SC_9	WAM97316	Stony Creek	*Velesunio* ‘sp. Lineage E’	50	25.2	26.6	32.9	16.3	0.53	0.95	0.66	0.33	0.61
YR2_1	WAM97317	Yule River	*Velesunio* ‘sp. Lineage F’	36.3	18.5	19.6	23.5	10.6	0.54	0.94	0.65	0.29	0.54
YR2_2	WAM97318	Yule River	*Velesunio* ‘sp. Lineage F’	37.5	20.7	22.2	25.3	10.5	0.59	0.93	0.67	0.28	0.47
YR2_3	WAM97319	Yule River	*Velesunio* ‘sp. Lineage F’	39	21.1	21.7	26.2	11.5	0.56	0.97	0.67	0.29	0.53
YR2_4	WAM97320	Yule River	*Velesunio* ‘sp. Lineage F’	59.6	33.5	35	38.5	19.2	0.59	0.96	0.65	0.32	0.55
YR2_5	WAM97321	Yule River	*Velesunio* ‘sp. Lineage F’	53.9	28.8	30.6	36.6	16.4	0.57	0.94	0.68	0.30	0.54
YR2_6	WAM97322	Yule River	*Velesunio* ‘sp. Lineage F’	50.4	25.5	27.9	36.1	16.4	0.55	0.91	0.72	0.33	0.59
1200	WAM97299	Anjammie Pool	*Velesunio* ‘sp. Lineage G’	76.2	35.7	38	46	23.8	0.50	0.94	0.60	0.31	0.63
1201	WAM97300	Stretch Lagoon	*Velesunio* ‘sp. Lineage G’	33.4	16	16.8	20.3	9.6	0.50	0.95	0.61	0.29	0.57
1202	WAM97301	Rocky Waterhole	*Velesunio* ‘sp. Lineage G’	81.5	41.8	44.1	57	26.3	0.54	0.95	0.70	0.32	0.6
1203	WAM97302	Rocky Waterhole	*Velesunio* ‘sp. Lineage G’	87	41	42.1	58.7	28.3	0.48	0.97	0.67	0.33	0.67
1205	WAM97304	Rocky Waterhole	*Velesunio* ‘sp. Lineage G’	79.7	39	39.6	54.7	22.5	0.50	0.98	0.69	0.28	0.57
1206	WAM97305	Rocky Waterhole	*Velesunio* ‘sp. Lineage G’	73	36.2	37.3	45.8	24.9	0.51	0.97	0.63	0.34	0.67
1207	WAM97306	Rocky Waterhole	*Velesunio* ‘sp. Lineage G’	78.2	37.4	39.6	53.4	23.8	0.51	0.94	0.68	0.30	0.6

*Note:* Five shell measurements were recorded: total shell length (TL), beak height (BH), maximum shell height (MH), long beak length (LBL) and shell width (W). Morphometric indices were maximum height index (MHI = MH/TL), beak height index (BHI = BH/MH), beak length index (BLI = LBL/TL), width–length index (WLI = W/TL) and width–height index (WHI = W/MH).

**TABLE A4:** Uncorrected pairwise distances for *16S*, *COI* and *28S* for the Velesunioninae sequences analysed in this study.

Species	*COI*			*16S*			*28S*		
	*N*	Intra	Inter	*N*	Intra	Inter	*N*	Intra	Inter
*Alathyria jacksoni* Baker	4	0.21–1.06	0.21–17.34	2	0.25–0.25	0.25–21.91			
*Alathyria jacksoni* Graf	1	NA	6.13–19.24	1	NA	3.54–21.86			
*Alathyria pertexta* NSW	1	NA	5.71–17.34	1	NA	1.26–21.91	1	NA	1.54–19.43
*Alathyria pertexta* QLD	1	NA	0.21–17.55	1	NA	0.25–21.91	1	NA	0.77–18.14
*Alathyria profuga*	2	0.63–0.63	6.13–19.66	2	0.25–0.25	2.78–22.22			
*Lortiella froggatti*	3	0.21–0.42	2.54–17.12	1	NA	5.54–22.42	2	0.26–0.26	1.03–19.33
*Lortiella rugata*	1	NA	2.54–17.34						
*Microdontia anodontaeformis*	1	NA	7.82–18.60	1	NA	4.53–20.35	1	NA	1.78–20.26
*Velesunio* ‘sp. Lineage A’	15	0.21–2.33	7.82–18.60	4	0.25–0.76	7.56–23.99	1	NA	1.03–18.39
*Velesunio* ‘sp. Lineage B’	5	0.42–3.81	4.23–19.66	3	0.25–0.50	0.76–21.91			
*Velesunio* ‘sp. Lineage D’	4	0.21–1.48	8.88–17.12	2	0.25–0.25	3.28–23.37			
*Velesunio* ‘sp. Lineage E’	6	0.21–2.11	7.19–18.18	4	0.25–0.50	4.52–22.11	3	0.25–0.51	1.53–19.74
*Velesunio* ‘sp. Lineage F’	1	NA	7.19–18.05	2	0.13–0.13	4.52–21.97	2	1.02–1.02	1.53–21.13
*Velesunio* ‘sp. Lineage G’	3	0.21–1.06	9.51–18.60	3	0.25–0.50	5.28–23.17	1	NA	0.77–18.39
*Velesunio ambiguus*	5	0.21–2.33	7.19–19.03	4	0.25–1.51	2.78–22.92	1	NA	3.35–17.62
*Velesunio angasi*	1	NA	7.61–17.55						
*Westralunio carteri*	4	0.21–0.63	5.07–19.03	4	0.25–0.50	3.01–22.42	1	NA	3.35–17.05
*Westralunio inbisi*	5	0.21–1.27	2.33–19.03	5	0.25–0.50	0.25–23.12	^a^	^a^	^a^
*Westralunio inbisi meridiemus*	4	0.21–1.48	2.33–19.66	2	0.50–0.50	0.25–23.12	^a^	^a^	^a^

*Note:* Values are presented as minimum–maximum. Blank rows indicate a lack of available sequence for that species.

Abbreviations: Inter, interspecific distance; Intra, intraspecific distance; *N*, number of haplotypes analysed.

^a^ All three *Westralunio* taxa shared the same haplotype for the *28S* fragment analysed.
